# Methods for Pluripotent Stem Cell Characterization: A Narrative Review

**DOI:** 10.7759/cureus.77362

**Published:** 2025-01-13

**Authors:** Fadoua Temsamani, Assia Agalit, Karima Idrissi Serhrouchni

**Affiliations:** 1 Laboratory of Histology, Embryology and Cytogenetics, Laboratory of Life and Health Sciences Research, Faculty of Medicine and Pharmacy of Tangier, Abdelmalek Essaâdi University, Tangier, MAR

**Keywords:** applications, characterization, characterization methodologies, pluripotent stem cells (pscs), standardization

## Abstract

Pluripotent stem cells (PSCs) have revolutionized medical research by offering significant advancements in regenerative medicine, disease modeling, and drug discovery. Several research and clinical studies have successfully generated PSCs but with varying characterization methodologies. Characterization methods are crucial to guarantee the safety and efficacy of PSCs in research and therapeutic applications. Therefore, there is a need for standardization of characterization methods to ensure their quality, reproducibility, and comparability across laboratories. In this review, the authors discuss the mandatory and non-mandatory assessments used for morphological, molecular, genetic, and functional PSC characterization. We will present their advantages and limits with the aim of providing insights for assessment optimization and helping facilitate the standardization of characterization methods.

## Introduction and background

The term stem cell was first introduced in the late 19^th^ century, and it has since mesmerized biologists and scientists with the idea of the ability of a single cell to generate all types of cells in the body [1]. Pluripotent stem cells (PSCs) are characterized by the ability to proliferate indefinitely due to their self-renewal capacity and have the potential to differentiate into cell types from all germ layers (ectoderm, mesoderm, and endoderm) [1-3]. Their proliferative and self-renewal capacity has made research on stem cells a promising and passionate field showing great promise in medical applications such as in regenerative medicine and transplant medicine, modeling human diseases, drug discovery and screening, and human developmental biology [3-5]. Clinical studies using PSCs are on the rise since they have been established in 14 countries, with Germany, Iran, and Sweden as latecomer countries in the field of PSCs-based therapies [6].

There are two types of PSCs: embryonic stem cells (ESCs) and induced pluripotent stem cells (iPSCs). Embryonic stem cells were first isolated in 1998 and are derived from the inner cell mass (ICM) of an embryo [7] and iPSCs were generated directly from somatic cells through a technology known as nuclear reprogramming [8,9]. While tissues derived from ESCs and iPSCs share similar molecular and functional characteristics [10], the use of ESCs requires the destruction of the blastocyte, which raises ethical concerns. Moreover, their safety concerns related to immune rejection and limited supply hinder their therapeutic applications. On the other hand, the use of iPSCs avoids ethical issues since they are derived from adult somatic cells, are easily and non-invasively obtained, and have a low risk of immune rejection, making them a favorable option for clinical applications and personalized therapies [3]. Nevertheless, despite the many advantages of iPSCs in comparison with ESCs, iPSCs represent some limitations related to their usage, including reprogramming efficiency, risk of tumorigenicity, and genetic and epigenetic instability [3]. Therefore, selecting the most appropriate type of PSC depends on a range of factors such as the goals of research or therapy, ethical considerations, and technical and practical requirements.

When developing new lines of PSCs, it is extremely crucial to carry out accurate characterization assays to define the new population of PSCs and to demonstrate their pluripotency and safety. Hence, several characterization tests recommended by the International Stem Cell Banking Initiative (ISCBI) need to be performed before the banking of new PSC lines and their usage in clinical and therapeutic applications [11]. These tests include pluripotency characterization, differentiation assays, genetic testing, determination of cell identity, gene expression profile, and sterility testing [11,12]. In June 2023, the International Society for Stem Cell Research (ISSCR) released a report including recommendations aiming to set standards for human stem cell line characterization and to improve transparency in research practices [13].

In summary, PSC characterization is an important step that must be carried out continually through the generation of PSCs for their banking or therapeutic use. However, protocol standardization is a must to guarantee the quality and reproducibility of PSCs in research and therapeutic applications in order to achieve a consensus. On that basis, we present in this review the most used methods for PSC characterization, including mandatory tests for release criteria and some methods that offer additional information on the safety and functionality of the PSC line. Furthermore, we will emphasize the assessments related to the morphological, molecular, genetic, and functional characterization of PSCs in order to identify areas for improvement and facilitate protocol standardization.

## Review

Characterization methods for PSC lines

Characterizing PSCs involves a multifaceted approach that examines the morphological, molecular, genetic, and functional aspects to ensure their safety and quality for research and clinical applications. Morphological characterization focuses on the cell's shape and overall size and structure of the colonies under a microscope [14]. This visual assessment provides initial insights into the pluripotency and differentiation status of the cells and colonies. Moving beyond the surface, molecular and genetic characterization involves analyzing the molecular features and genetic properties of PSCs by performing various tests. These tests help identify key pluripotent markers and detect specific genetic modifications confirming the pluripotent state, identifying genetic aberrations and differentiation potential [2,15]. Several techniques utilized are either mandatory methods for release tests or to provide additional information on PSCs for molecular and genetic characterization (Table 1) [15,16]. Finally, functional characterization assesses the ability of PSCs to differentiate into various cell types and contribute to tissue formation in vivo. The methods include differentiation assays, embryoid body formations, and teratoma formation [15,17].

**Table 1:** Summary of mandatory and ‘for information only’ methods used for PSCs characterization NA: no release criterium available; qRT-PCR: quantitative reverse transcription polymerase chain reaction; SNP: single nucleotide polymorphism; CGh: comparative genomic hybridization; PSC: pluripotent stem cells

Characteristics	Release criteria methods	For information only methods
Morphology	Photography	NA
Pluripotency	Flow cytometry	Immunocytochemistry, qRT-PCR, alkaline phosphate
Genetic stability	Karyotype analysis	SNP analysis, CGH array
Differentiation	Embryoid bodies formation/directed differentiation	Teratoma formation

Morphological characterization

Human PSCs (hPSCs) grown on feeders show a particular and distinct morphology where the cells show a small and round shape with a large nucleus and exhibit a scant cytoplasm and a prominent nucleolus. The colonies are characterized by a round and flat shape and are tightly organized, exhibiting well-defined edges [2,14,18]. Wakao et al. described in a detailed study the criteria to determine iPSC colonies as exhibiting a single nucleolus, a nucleus-to-nucleolus ratio of about 2.19, and a nucleus-to-cytoplasm ratio of about 0.87, where the cells are comprised in a single layer at a density of about 5900 cells/mm² and each cell is around 43.5 mm² [19]. Culture conditions influence cell and colony morphology; therefore, stem cell labs and banks should provide representative images of undifferentiated and differentiated cells, along with undesirable differentiation features for researchers to compare with their own cultures for learning purposes [12, 15].

To our knowledge, there is no standardized type of microscope that needs to be used to evaluate the morphological characteristics of PSCs. However, the morphological observation of PSCs is usually performed using a range of optical microscopy techniques, each providing unique insights into cellular structure and characteristics. Brightfield microscopy is often used to observe unlabelled, vital, and thin samples [20] and can provide only the general morphology of living cells in culture [21]. Therefore, for more detailed imaging and better evaluation of distinguishing characteristics of stem cells [20], phase-contrast microscopy is the most used tool to evaluate the morphological characteristics of PSCs [18, 21-25]. It offers enhanced contrast without staining the cells, thereby yielding detailed images of live PSCs [26]. It generates images by using optical systems to convert light wave variations passing through the cell and its surroundings to a visible image with differences in contrast [26, 27]. Phase-contrast microscopy offers sustained monitoring of live cell proliferation processes such as cell cycle, cell migration, and differentiation by time-lapse recording images [27]. However, biological experiments that require the generation of large quantities of image data can make the manual analysis work-intensive, lengthy, and prone to error [27]. Moreover, the bright halo effects and shade-off artifacts can make image analysis difficult [28]. Therefore, computer-assisted microscopy has made image analysis much easier and more accurate [28]. Furthermore, developed segmentation procedures that provide cell detection and tracking can offer artifact-free images, making image analysis and interpretation more efficient [28,29].

Nevertheless, fluorescent microscopy is employed to gain more details about specific cellular structures of PSCs. It’s a sensitive method that uses specific light-emitting probes or dyes to label live or fixed cells, providing high contrast and resolution of subcellular structures [20,30]. This technique is employed to evaluate PSC characteristics during differentiation, to identify key markers of pluripotency, or to indicate cell health and function [20]. For the highest resolution, electron microscopy is employed to provide detailed information on the cellular ultrastructure, revealing proteins to small membrane subdomains of cell morphology [31]. Different techniques are used to evaluate the molecular characterization of PSCs.

Molecular characterization

Pluripotency is the ability of a single cell to differentiate into all the mature cell types of the body. Both ESCs and iPSCs are renowned for expressing a set of markers validating their pluripotent status and are important for maintaining their undifferentiated state. The International Stem Cell Initiative (ISCI) identified a core set of markers associated with maintaining pluripotency in a large group of human embryonic stem cell (hESC) lines, including NANOG, OCT4, TDGF1, DNMT3B, GABRB3, and GDF3 as intracellular markers and the glycolipid antigens SSEA3 and SSEA4 and the keratan sulfate antigens TRA-1-60 and TRA-1-81 as surface markers [11]. There are several downregulated markers expressed in hPSCs [2], among them, SSEA1, which is expressed only during hPSC differentiation [15]. Different techniques are used to evaluate the molecular characterization of PSCs.

Flow Cytometry

Flow cytometry is an immunophenotyping method that has been intensively used for identifying various cell markers and is considered a mandatory technique for PSC identification and characterization [12, 15]. It utilizes different types of fluorescent reagents to measure the physical and chemical properties of single cells in solution while they flow through a single or multiple lasers [32,33]. It can be performed on a variety of tissues and provides parameter analysis of cell size and granularity [32]. Flow cytometry can even measure multiple parameters of isolated cells, such as viability, cell proliferation, intracellular cytokine production, and cell cycle phase [34], and detect intracellular markers [16]. Fluorescence-activated cell sorting (FACS) is an updated version of flow cytometry that offers qualitative and quantitative stem cell analysis by identifying cell populations based on granularity, fluorescence, size, and charge of the targeted cell. It also offers constant, accurate, and highly purified isolation of single cells from assorted mixtures of cell suspension or live tissue and can be used for more downstream analysis [35]. Flow cytometry is a fast and robust method that provides quantitative results, making it easier to compare results with other laboratories. However, it requires trained personnel, is limited to markers used for characterization, and has a risk of contamination.

This method enables quantification of cell surface markers (SSEA3, SSEA4, TRA-1-60, TRA-1-81) that are easily accessible by fluorescently labeled antibodies and can detect the internal markers (OCT4, SOX2, NANOG) by an additional step that requires fixation [15,16,36]. As for hPSC markers, it is recommended to use a combination of surface markers and internal markers for better characterization of hPSCs [15]. According to ISCBI, the acceptance criteria of hPSC marker characterization by flow cytometry is at least ≥70% of hPSCs expressing pluripotency markers and ≤10 of hPSCs expressing the surface marker SSEA-1 [37]. Nonetheless, ISSCR does not consider these molecular markers as uniquely expressed by hPSCs but rather can be expressed by other stem cells that have lost the ability for differentiation. Therefore, these markers cannot be used for hPSCs characterization only without functional evidence of the pluripotent status [13].

*Immunocytochemistry * 

Immunocytochemistry is a laboratory method used to visualize the localization of a specific antigen in tissue and cells [38,39]. It can use primary antibodies conjugated with fluorophore groups to bind specifically with the antigen or use primary and secondary antibodies in which the secondary antibody is conjugated with fluorophore groups. This allows antigen localization and visualization under a fluorescence microscope [38]. Immunocytochemistry provides a general assessment of the expression of specific cell markers within a colony or culture [40]. It detects both surface markers of pluripotency (TRA-1-60, TRA-1-81, SSEA3, SSEA4) and intracellular markers (OCT3/4, SOX2, NANOG) [15]. However, immunostaining of intracellular markers requires a step of fixation [2]. It is recommended to use a combination of intracellular and surface markers for characterization of hPSCs [15]. Nevertheless, immunocytochemistry analysis is not mandatory for hPSC marker characterization but is rather used to provide additional information only on morphology and specific marker localization [12,41]. Nonetheless, immunocytochemistry is recognized by ISSCR as an immunophenotyping method for the assessment of cell markers [13] and is used for hPSCs characterization by many authors.

The immunocytochemistry method is characterized by a high level of sensitivity for localizing the presence of an antigen in a cell, has a fast processing time, and is low cost. However, this technique has a moderate to high level of selectivity, which determines the accuracy of the results [38]. Moreover, interpretation can be difficult, making comparison with other results more challenging, and it requires trained personnel and strict evaluation criteria [15]. Western blot assay can be used as a complementary assessment to immunocytochemistry to further evaluate and quantify hPSC characteristics [42,43].

Quantitative Reverse Transcription Polymerase Chain Reaction

Real-time polymerase chain reaction is a technique generally used for gene expression characterization and to test for genetic diseases in different types of tissues and cells; RT-PCR generates cDNA from mRNA by combining a reverse transcription reaction with the conventional PCR-based amplification [44]. It’s a sensitive method and requires a very small amount of sample and can detect gene expression from an isolated cell [44]. As for quantitative PCR (qPCR), it is based on the conventional PCR method, which is utilized to amplify and quantify a target DNA [45]. Fluorescent detection systems in qPCR are employed to allow analysis during amplification and simultaneous DNA quantification [45]; qPCR is a widely used technique for nucleic acid detection and quantification with precision and is sensitive for analyzing gene expression [46]. However, qPCR-reproducing data can be challenging, thus the need for an optimized designated approach for gene expression analysis [46].

Both qPCR and RT-PCR can be combined to quantify the gene of interest [45]; qRT-PCR can be utilized to determine the expression of intracellular and cell surface markers of hPSCs [47]. This method is preferable for providing quantitative and semi-quantitative results and is highly beneficial when antibodies for hPSCs characterization are not available [2]. According to ISSCR, qPCR assays are primarily utilized for quality control analysis of iPSCs cultures and for detecting genetically variant cells. Nonetheless, qRT-PCR is used for clinical-grade hPSCs characterization of pluripotency markers by many authors; PCR-based methods are used for testing hPSCs derivatives, and newly developed PCR-based technologies (like droplet digital PCR) can be used to improve the effectiveness and precision of assessing iPSC characteristics and their products [16].

Alkaline Phosphatase (AP)

Alkaline phosphatase staining is often performed to evaluate hPSCs phenotype. The high level of AP is an indicator of pluripotent and undifferentiated stem cells [48]; AP staining is a quick and sensitive test that relies on the use of a chromogenic substrate for visualizing PSC-positive colonies under a microscope [49]. Yet AP doesn’t reflect the stemness of stem cells but rather the process of differentiation, and it can be expressed in other cell types instead of stem cells only [48]. Therefore, these limitations must be considered while interpreting AP staining results. 

Genomic characterization

Stem cells are prone to genetic alterations in cultures [13]. Long-term cultures have shown acquired genetic mutation in hESCs and have been asserted that iPSCs are likely more prone to genetic changes than other hPSC populations [41]. These genetic changes can cause alterations or loss of hPSCs functional characteristics and lead to a transformed tumorigenic state [37]. Therefore, it is crucial to routinely monitor the presence of genetic changes and to identify chromosomal, subchromosomal, or nucleotide alterations for the safe usage of stem cell therapy [13,16]. There are various methods used to assess the genetic stability of hPSCs.

Karyotype

Karyotype analysis by G-banding is the most widely used method for genomic stability evaluation and can detect changes in chromosomal number and structure aberrations [13,50]. This method is considered a mandatory assessment to routinely evaluate hPSCs' genetic stability [12,37]. This technique utilizes Giemsa dye to stain chromosomes at metaphase. The staining results in banding patterns that allow the identification of chromosomal abnormalities under a microscope [2, 50]. The requirement of G-banding analysis is to use chromosome counts of 20 metaphases and analysis of banding patterns in a minimum of eight metaphases [12]. This analysis confirms that the cells are karyotypically normal [15]. If the cells show abnormalities, repeating the test is recommended for findings confirmation since these hPSCs lines can be deemed abnormal [12]. Also, it is recommended to repeat genetic evaluation every 10 passages, after major culture bottlenecks, or if any alterations are shown in stem cell traits [13].

G-banding is a standardized method that can provide a clear visual of the entire chromosome in an isolated cell in a single assay and can detect aneuploidies and balanced translocations. However, it has a low resolution for detecting small genetic variations, can be time-consuming, and needs specialist skills for accurate interpretation [13]. Spectral karyotyping and fluorescent in situ hybridization (FISH) are alternative techniques relying on the use of fluorescently labeled probes that bind to specific regions of chromosomes, enabling the identification of structural anomalies such as translocations or duplications [51]. Furthermore, spectral karyotyping offers enhanced resolution compared to conventional karyotyping, making it a better alternative to conventional karyotyping [50].

Single Nucleotide Polymorphism (SNP) Array 

Single nucleotide polymorphism array is currently a fundamental method for clinical cytogenetics diagnostics used for detecting genomic aberrations [41]; SNP array enables copy number variations (CNVs) detection, such as subchromosomal duplications and deletions, identifying triplication, mosaicism, and homozygous loss [51,52]. It is based on complementary labeled probes binding to particular SNPs. The labeled probes generate fluorescent signals indicating the presence or absence of SNPs. The signal intensity when detected helps identify genetic variants with a higher resolution than G-banding and can even detect genomic abnormalities in normal karyotyped cells [52]. hPSCs contain high rates of subchromosomal CNVs compared to other somatic cells [53]. Therefore, SNP arrays can be used to conduct genome-wide screens for detecting chromosomal aberrations for hPSCs characterization [53]. However, SNP arrays cannot detect balanced chromosomal translocations and inversions [15,52], nor can they identify mosaic genetic changes in samples only once they contain <20% variants [52]. Therefore, SNP array analysis is only recommended to be used to provide additional information for hPSCs characterization and preferably to be combined with karyotype analysis to enable a comprehensive assessment of hPSCs genetic integrity [13,15,54].

*Array Comparative Genomic Hybridization* *(aCGH)*

Comparative genomic hybridization (CGH) is an adaptation of the FISH technique in which the fluorescent intensity from a sample of genomic DNA is compared to that from a reference genome to help identify the copy number of the assessed sequence. Array CGH is the combination of CGH and microarray technology allowing CNV detection in a single experiment [55]. Array CGH is increasingly used to evaluate the genetic integrity of hPSC cultures [56] and is part of genome-characterizing methods recommended for assessing the genetic stability of clinical-grade hPSCs [13]. Array CGH allows genetic detection with a higher resolution than conventional karyotyping [15] and can detect small amounts of CNVs as small as a few hundred bases [55]. On another hand, this method cannot detect low-level mosaicism nor balanced chromosomal rearrangements [57], and interpretation of detected anomalies can be quite challenging as their clinical consequences are not known yet [55]. Therefore, combining aCGH and G-banding is the best recommended approach to assess the genetic integrity of analyzed cells, and in the imminent future, advanced high-throughput sequencing strategies may offer an appealing strategy for routine monitoring of genomic aberrations [57].

Functional characterization

To characterize an hPSC line as pluripotent, it has to be able to show the capacity of differentiation onto cells making the three germ layers: ectoderm, definitive endoderm, and mesoderm [13]. Pluripotency used to be demonstrated by the ability of cells to form germline chimeras when characterizing mouse ESCs. However, for ethical reasons, hPSC pluripotency is being assessed by other surrogate assays to confirm the pluripotent status [13]. Different strategies are employed to evaluate the capacity of differentiation of hPSCs lines. 

Embryoid Bodies Formation

Spontaneous differentiation is the most direct method used to assess the differentiation potential of hPSCs [15]. When cultured in suspension without fibroblast growth factor (FGF), they tend to form self-organizing aggregates called embryoid bodies (EB) [58,59]; hPSCs growing as EB should differentiate into cells of the three germ layers and be analyzed to confirm their differentiation status [15,50]. The most used approach to generate EB is by simply detaching PSC colonies using the enzymatic treatment and culturing them in media in Petri dishes or low-adherence plates [58]. Embryoid bodies can also be produced by seeding single hPSCs in hanging drops, U- or V-shaped bottom wells, or by using 3D bioprinting and hydrogels [58]. Afterward, fully grown EB can either be used for random differentiation or directed differentiation [2]. Embryoid bodies can be kept in suspension or transferred and attached to an adherent-coated surface in random differentiation. They are grown without FGF for over seven to 21 days [2].

Immunocytochemistry or RT-PCR can be used to confirm trilineage differentiation by analyzing various differentiation markers of EB [2, 15]. The most common markers used include beta-III-tubulin (TUJ1) for ectoderm, alpha-fetoprotein (AFP) or SOX17 for endoderm, and smooth muscle actin (SMA) for mesoderm [2, 15, 60]. Currently, there is no standard method for performing the spontaneous differentiation assay of EB since other factors like different methods, culture media, and EB sizes can affect the trajectory of differentiation [2]. Embryoid body generation assay is a time- and cost-efficient method that reflects the preferential tendencies of hPSCs to differentiate into one specific germ layer more than the others. However, since EB are 3D aggregates of different cell types, they can be quite challenging for analysis and quantification by immunocytochemistry, leading to ambiguous results [15].

Directed Differentiation

Human pluripotent stem cells can be characterized by directed differentiation to assess the potential of trilineage pluripotency. hPSCs are exposed to specific growth factors and culture media conditions to promote differentiation in each germ layer [56]. According to ISSCR, it is recommended to evaluate the expression of two or three specific markers of each germ layer to be able to assess the functional characterization of hPSCs [13]. Generally, marker detection and quantification can be assessed by immunocytochemistry [15] and RT-PCR, flow cytometry, and in situ hybridization [2]. The directed differentiation can be accomplished by using kits to generate a mix of precursor cells from a particular lineage. The differentiated cells are usually in a monolayer and more homogenous, making further analysis much easier [15,61,62]. Kits are available and time efficient and provide a straightforward workflow; however, they may not be able to yield a pure population of a specific cell type. Yet with in-house methods of differentiation, it is possible to generate specific cell types, but the process can be time-consuming and costly [15,62]. Currently, there is no standardized protocol used for directed differentiation of hPSCs, as robust protocols are not very common, making obtaining consistent results challenging [15]. Directed differentiation and EB formation can be assessed by commercialized gene expression assays Pluritest and hPSCs ScoreCard. These methods can be used to provide additional information for functional pluripotency evaluation [41].

Teratoma Formation

Teratoma assay used to be the most crucial method for hPSC pluripotency assessment [61,63]. The assay consists of injecting immunodeficient mice with hPSCs. The injected pluripotent cells will then differentiate and form a teratoma comprising tissues from all three germ layers [50]. Differentiation is confirmed by evaluating the differentiation markers by immunohistochemistry [50]. Teratoma assays can be valuable for indicating the malignant potential of the injected hPSCs line and can further evaluate the maturation and histogenesis of the hPSC-derived differentiated cells. However, this method has many restrictions and is not recommended to be used as a routine assessment of hPSCs functional characterization [13,61]. Moreover, teratoma formation is time-consuming and expensive. It requires a large number of experimental animals and can be restricted due to animal welfare. The production of teratomas and their assessment necessitates qualified experts, as results can be inconsistent and difficult for quantification, making data comparisons from different laboratories difficult [61,63]. In vitro 3D assays or organoids can be used to evaluate the morphogenesis and histogenesis of hPSCs [13]. Combining EB formation, directed differentiation, and gene expression assays can be used to efficiently characterize hPSC pluripotency and substitute the adoption of teratoma assays [13,37].

Overview of the Characterization Methods of hPSCs

Characterization of hPSCs is a crucial process to evaluate their properties, ensuring their quality and safety for further research and therapeutic applications. It consists of various approaches that aim to assess hPSCs on different levels. Morphological characterization utilizes various microscopes, particularly phase-contrast microscopes, to evaluate the shape, size, and colony structures to identify the status of the cells. Meanwhile, molecular characterization aims to evaluate cell pluripotency and genetic stability. Pluripotency characterization is performed by immunophenotyping methods to evaluate specific cell markers such as flow cytometry and immunocytochemistry. Furthermore, gene expression analyses such as qRT-PCR and alkaline phosphatase immunostaining are also employed to identify PSC pluripotency status. As for genetic characterization, the assessment of genetic changes in hPSCs is employed to detect chromosomal and sub-chromosomal aberrations and identify nucleotide changes. It is performed by several methods, such as karyotyping, SNP array, and aCGH profiling. Lastly, functional characterization aims to evaluate the ability of hPSCs to differentiate into lineage-specific cells and is often assessed by directed differentiation and/or embryoid body formation and rarely by teratoma formation. Immunophenotyping methods and gene expression analysis methods can furthermore be employed to evaluate lineage-specific genes for differentiation characterization. Characterization methods are summarized with each their status of acceptance criteria, advantages, and limits (Table 2).

**Table 2:** Summary of hPSCs characterization methods NA: no release criterium available; qRT-PCR: quantitative reverse transcription polymerase chain reaction; RT: reverse transcriptase; SNP: single nucleotide polymorphism; CNV: copy number variation; CGH: comparative genomic hybridization; PSC: pluripotent stem cells; hPSCs: human pluripotent stem cells

Characteristics	Methods	Acceptance criteria	Advantages	Limits
Morphology	Phase contrast microscopy	NA	Live cell visualization, label-free, non-invasive, recording time-lapse images, analyze cell migration and behavior	Halo effect and shade-off, limited resolution, not ideal for thick specimens
Pluripotency	Flow cytometry	Mandatory	Qualitative and quantitative assay, high precision, multiparametric analysis cell sorting, analysis of cell heterogeneity, viability testing; used for downstream analysis	Expensive equipment and maintenance, requires trained personnel, limited to used markers, possibility of compromised fluorophores, limited information of cellular structures, risk of contamination
	Immunocytochemistry	For information only	Fast processing time, high specificity for antigen localization, allows antigen localization, cell morphology preservation, applicable to different cell types	Quantitative limitations, require validated antibodies, necessitate strict evaluation criteria, require microscopy expertise
	qRT-PCR	For information only	Low cost, fast results, high specificity and sensitivity, quantitative and semi-quantitative gene expression, high throughput, small amounts of RNA	Risk of RNA degradation, RT step can increase time, price, and contamination potential; non-recyclable product; less flexibility for primer and probe selection; increased risk of false negatives for new pathogen detection
	Alkaline phosphatase	For information only	Quick and easy test, wide range of applications, useful for monitoring differentiation in stem cells	Possible false results, non-specific, expression variability
Genetic stability	Karyotyping	Mandatory	Low cost, wide availability, identification of numeric and structure chromosomal aberrations, analysis of the whole genome	Time-consuming, low resolution (5 – 10 Mo), requires plasma cells during metaphase
	SNP	For information only	Fast and easy to perform, cost-effective, detects genomic aberrations, detects CNVs and SNPs, mosaicism and homozygous loss identification, high throughput, highly specific probes	Enables the detection of balanced translocations or inversions, low resolution for detecting low-level CNVs, determines only known genomic aberrations
	Array CGH	For information only	Detects constitutional chromosomal aberrations, detects CNVs in a single assay, conducted on whole genome, high resolution, detects small amounts of CNVs	Expensive, enables the detection of balanced rearrangements, enables the detection of ploidy changes and mosaicism, difficulty of interpretation
Differentiation	Embryoid bodies formation	Mandatory	Time and cost-efficient, mimics early stages of embryonic development, PSC differentiation assessment, trilineage differentiation, useful for therapeutic applications	Labor intensive and requires standardization, viability issues, analysis and interpretation can be challenging, differentiation variabilities, stringent conditions for culture and differentiation
	Directed differentiation	Mandatory	Rapid process and straightforward workflow by using kits, assess trilineage differentiation / Useful for therapeutic applications	Labor intensive, challenging for obtaining consistent results, incomplete or variability differentiation, can yield low purity of cell types, needs standardization
	Teratoma	For information only	Assessment of pluripotency, studying stem cell behavior and differentiation, useful for testing stem cell therapies	Labor intensive, time consuming and expensive, ethical issues, risk of tumor formation, requires a large number of experimental animals, requires qualified experts, inconsistent and difficult data analysis

## Conclusions

Human pluripotent stem cells are of paramount importance across multiple disciplines, particularly in regenerative medicine, disease modeling, and drug discovery. Characterization methods of hPSCs offer a comprehensive evaluation of their characteristics, providing safe and effective clinical usage. In this review, we presented the mandatory assessments and methods used to provide additional information for the characterization of hPSCs. We furthermore summarized their advantages and limits to help identify suitable methods for characterization and encourage the development of new techniques.

Nevertheless, there is a need for characterization protocol standardization to ensure consistent reproducibility and reliability of hPSCs in research and clinical applications. The integration of emerging technologies can help enhance the specificity and sensitivity of hPSCs characterization. Therefore, these technologies can help optimize hPSCs characterization protocols and make method standardization less challenging, further enhancing the potential of hPSCs advancements in research and medical applications.
